# Subcutaneous C1 inhibitor for prevention of attacks of hereditary angioedema: additional outcomes and subgroup analysis of a placebo-controlled randomized study

**DOI:** 10.1186/s13223-019-0362-1

**Published:** 2019-08-28

**Authors:** H. Henry Li, Bruce Zuraw, Hilary J. Longhurst, Marco Cicardi, Konrad Bork, James Baker, William Lumry, Jonathan Bernstein, Michael Manning, Donald Levy, Marc A. Riedl, Henrike Feuersenger, Subhransu Prusty, Ingo Pragst, Thomas Machnig, Timothy Craig, for the COMPACT Investigators

**Affiliations:** 1 Institute for Asthma and Allergy, 2 Wisconsin Cir #250, Chevy Chase, MD 20815 USA; 2 UC San Diego School of Medicine, 9500 Gilman Dr., Mail code 0732, La Jolla, CA 92093-0732 USA; 3 Addenbrooke’s Hospital, Cambridge, CB2 0QQ UK; 4 Ospedale Luigi Sacco/U.O. Medina Generale, Via G.B. Grassi, 74, 20157 Milan, Italy; 5 Department of Dermatology, Johannes Gutenberg University Mainz, Langenbeckstr. 1, 55131 Mainz, Germany; 6 Baker Allergy, Asthma & Dermatology Research Center, LLC, 9495 SW Locust, Portland, OR 97223 USA; 7 AARA Research Center, 10100 N Central Expressway, Suite 125, Dallas, TX 75231 USA; 8 Bernstein Clinical Research Center, LLC, 8444 Winton Road, Cincinnati, OH 45231 USA; 9 Medical Research of Arizona, 7514 E Monterey Way, Suite-1A, Scottsdale, AZ 85251 USA; 10 705 West La Veta Avenue, Suite 101, Orange, CA 92868 USA; 11 University of California-San Diego School of Medicine, 8899 University Center Lane, Suite 230, La Jolla, CA 92122 USA; 12 CSL Behring GmbH, Marburg, Germany; 13 Department of Medicine and Pediatrics, Penn State University Allergy, Immunology and Respiratory Research, 500 University Drive H041, Hershey, PA 17033 USA

**Keywords:** C1-esterase inhibitor protein, C1-INH (SC), COMPACT study, HAEGARDA^®^, Hereditary angioedema, Long-term prophylaxis, Replacement therapy, Subcutaneous

## Abstract

**Background:**

Hereditary angioedema (HAE) is a debilitating disorder resulting from C1-esterase inhibitor (C1-INH) deficiency. In the COMPACT phase 3 study the prophylactic use of a subcutaneous C1 inhibitor (C1-INH [SC], HAEGARDA^®^, CSL Behring) twice weekly significantly reduced the frequency of acute edema attacks. Analysis of treatment effects by subgroups, onset of effect, and other exploratory analysis have not been reported.

**Methods:**

This is a post hoc exploratory analysis on data from the randomized, placebo-controlled COMPACT study. 90 patients with C1-INH-HAE were randomized to 1 of 4 treatment sequences: C1-INH (SC) 40 or 60 IU/kg of body weight twice weekly for 16 weeks, preceded or followed by a placebo period. The pre-specified primary efficacy endpoint was the time-normalized number of HAE attacks, and pre-specified secondary efficacy endpoints were the percentage of patients with a certain treatment response (≥ 50% reduction on C1-INH (SC) versus placebo in the time-normalized number of attacks) and the time-normalized number of use of rescue medication. Pre-specified exploratory endpoints included severity of attacks, alone and combined with rescue medication use. Post hoc analyses included exploration of onset of effect and clinical assessment of patients with < 50% of response.

**Results:**

Subgroup findings by various patient characteristics showed a consistent preventive effect of C1-INH (SC). In a post hoc analysis of attacks, the onset of the preventive effect within the first 2 weeks after treatment initiation in COMPACT showed that 10/43 patients (23%) experienced attacks of any severity with 60 IU/kg versus 34/42 patients (81%) with placebo. The need for rescue medication was tenfold lower with 60 IU/kg (35 treated attacks) versus placebo (358 treated attacks). A qualitative analysis of the 4 patients treated with 60 IU/kg and with < 50% reduction of attacks demonstrated a reduction in severity of attacks, rescue medication use, and symptom days which was considered a clinically meaningful treatment effect.

**Conclusions:**

C1-INH (SC) prophylaxis demonstrated a preventive treatment effect with evidence of benefit within 2 weeks. A consistent treatment effect at recommended C1-INH (SC) dosing was evident in all subgroups of patients with type I/II HAE and by various measures of disease and treatment burden.

*Trial registration* EU Clinical Trials Register, 2013-000916-10, Registered 10 December 2013, https://www.clinicaltrialsregister.eu/ctr-search/trial/2013-000916-10; ClinicalTrials.gov Register, NCT01912456, Registered 31 July 2013, https://clinicaltrials.gov/ct2/show/NCT01912456.

## Background

Hereditary angioedema (HAE) is a painful, debilitating, and potentially fatal disorder characterized by recurrent episodes of swelling of various parts of the body [1–4]. HAE is an autosomal dominant disorder resulting from a quantitative deficiency (referred to as type I) or dysfunction (referred to as type II) of C1-esterase inhibitor (C1-INH) protein, leading to an increase in bradykinin levels and thus increased capillary permeability, manifesting as attacks of edema [5–7].

C1-INH replacement therapy has been used for many decades for the management of HAE symptoms and is recommended as a first-line acute treatment for HAE symptoms and for the long-term prophylaxis of attacks [8]. Routine prophylaxis with intravenous (IV) long-term C1-INH replacement therapy (C1-INH [IV]) has been used since 2008 based on the outcomes of the CHANGE study [9]. However, with C1-INH (IV) patients may still experience attacks, and some patients have venous access challenges [10–12]. A highly-concentrated human plasma-derived C1-INH for subcutaneous injection (C1-INH [SC], HAEGARDA^®^, CSL Behring) has been approved by regulatory agencies for long-term prevention of HAE attacks in adolescents and adults based on the phase 3 COMPACT study [5, 13]. The currently approved C1-INH (SC) dose of 60 IU/kg of body weight (bw) administered subcutaneously twice weekly reduces the attack rate by a median of 95% and the use of rescue medications is reduced by a median of 100% [5]. Two doses (40 and 60 IU/kg bw) tested in the COMPACT study were well tolerated and the most commonly reported side effects were mild injection site reactions, which occurred similarly during both active and placebo administrations.

Here we report pre-specified as well as post hoc exploratory findings of the placebo-controlled COMPACT study to further characterize the prophylactic treatment effects of C1-INH (SC) in type I or II HAE patients.

## Methods

### Study design

The study design, patient inclusion and exclusion criteria, treatments, clinical assessments, and key outcome measures and statistics have been described previously [5, NCT01912456]. In brief, COMPACT was an international, prospective, multicenter, randomized, double-blind, placebo-controlled, incomplete crossover, phase 3 dose-ranging study. Patients entered a run-in period of up to 8 weeks. If the patients experienced at least 2 HAE attacks during a consecutive 4-week period or at least 1 HAE attack during the first 2 weeks of the run-in period, they were randomized 1:1:1:1 via an interactive response technology system to receive C1-INH (SC) 40 IU/kg for an initial 16-week treatment period (TP1) followed by placebo for a second 16-week treatment period (TP2) or vice versa (i.e., placebo in TP1 and C1-INH [SC] in TP2) in a crossover design, or 60 IU/kg followed by placebo or vice versa for two 16-week periods.

### Patients

Eligible patients were at least 12 years of age, with a clinical and central laboratory diagnosis of type I or II HAE, who experienced at least 4 attacks over a 2-month period within 3 months before screening.

### Treatments

The C1-INH (SC) study product is a highly-concentrated (500 IU/mL), human plasma-derived, pasteurized and nanofiltered preparation, provided as a lyophilized powder to be reconstituted with sterile water for injection. C1-INH (SC) or placebo was self-administered twice weekly in the abdominal area, unless the investigator thought an alternative site was clinically more appropriate. Patients were permitted to use C1-INH (IV), icatibant, ecallantide, or fresh-frozen plasma as rescue medication for on-demand treatment of attacks or for pre-procedural prophylaxis.

### Outcome measures

Patients recorded their HAE symptoms and their use of rescue medications daily in an electronic diary. At each study visit, the investigator reviewed the diary and recorded attacks and their details on an electronic case report form.

The pre-specified primary efficacy endpoint was the time-normalized number of attacks of angioedema. Secondary pre-specified efficacy endpoints were the percentage of patients who had a certain response (≥ 50% reduction in the time-normalized number of attacks with CSL830 as compared to placebo) and the time-normalized number of rescue medication use. The numbers of attacks and uses of rescue medication were normalized for the number of days that the patient received the corresponding treatment.

Findings on the primary, secondary, and selected exploratory endpoints as well on health-related quality of life endpoints have been reported previously [1, 5]. The COMPACT study also assessed exploratory endpoints which included number of days of angioedema symptoms, severity of attacks, and percentage of patients in whom the number of attacks was reduced to less than 1 attack per 4-week period from 1 attack or more per 4-week period with placebo. The results of those endpoints have been reported previously [5].

We present a post hoc analysis on the number of HAE attacks that required treatment with rescue medication, assessed if a preventive effect within 2 weeks of treatment initiation was evident, and determined the percentage of patients who experienced HAE attacks in the first 2 weeks of treatment initiation. For the pre-specified primary efficacy analysis of the COMPACT study, attacks occurring within the first 2 weeks after treatment initiation were not included, since steady-state conditions of C1-INH functional activity were not expected to be reached beforehand. Patients treated with 60 IU/kg who were found to have a < 50% reduction in the time-normalized number of HAE attacks versus placebo were assessed in further detail.

Other exploratory endpoints not yet reported were time-normalized sum of severity scores recorded for every day of reported HAE symptoms, time-normalized sum of severity scores recorded for every day of reported HAE symptoms in combination with the number of rescue medication use, and duration of HAE attacks per patient (for definition see Table 1).

**Table 1:** Definition of exploratory endpoints

Exploratory endpoint	Method of calculation
Time-normalized sum of symptom severity scores	For every day of recorded HAE symptoms, the patient was to grade the severity of each symptom as mild = 1, moderate = 2, or severe = 3 The time-normalized sum of these severity scores for every day of recorded symptoms per patient was calculated as $\frac{{{\text{Sum}}\,{\text{of}}\,{\text{severity}}\,{\text{scores}}\,{\text{for}}\,{\text{every}}\,{\text{day}}\,{\text{of}}\,{\text{recorded}}\,{\text{HAE}}\,{\text{symptoms}}\, ( {\text{yes)}}\,{\text{in}}\,{\text{treatment}}\,{\text{period}}}}{{{\text{Number}}\,{\text{of}}\,{\text{days}}\,{\text{with}}\,{\text{entered}}\,{\text{HAE}}\,{\text{symptoms}}\, ( {\text{yes/no)}}\,{\text{in}}\,{\text{treatment}}\,{\text{period}}}}$
Time-normalized sum of symptom severity scores combined with rescue medication use	The time-normalized sum of symptom severity scores for every day of recorded HAE symptoms combined with the number of uses of rescue medication was defined and calculated as follows Rescue medication was coded as 1 if no rescue medication was taken and 2 if any rescue medication was taken The symptom severity score for each day of recorded HAE symptoms was multiplied by the rescue medication code to give a combined value The combined values from all days of recorded HAE symptoms during the treatment period were summed The summed value was divided by the number of days of entered HAE symptoms (yes/no) in the treatment period

HAE: hereditary angioedema

### Subgroup analyses

A subgroup analysis was pre-specified for both endpoints, time-normalized number of HAE attacks and percentage of responders. Subgroup analyses were performed only if there were ≥ 5 patients in a subgroup.

The pre-specified subgroups were: region (US, non-US), sex, race (American Indian or Alaska Native, Asian, Black or African American, Native Hawaiian or other Pacific Islander, White, other), age class (12 to < 17 years, 17 to < 65 years, 65 years or older), HAE attack location (facial, peripheral, laryngeal, thoracic, abdominal, urogenital, other), mucosal and non-mucosal attack location, use of oral prophylaxis for treatment of HAE during the study, received prophylactic medication (plasma-derived C1-INH prophylaxis [Cinryze^®^/Berinert^®^/C1-INH not otherwise specified], oral prophylaxis [androgens or progestins, tranexamic acid or aminocaproic acid], no C1-INH or oral prophylaxis reported) for at least 1 month during the 3 months before screening.

### Statistics

All efficacy analyses were performed on the intention-to-treat (ITT) population, which comprised all randomized patients. Results on the exploratory endpoints were summarized descriptively by treatment. For the subgroup analyses of the pre-specified primary endpoint, least squares means were estimated with 95% confidence intervals using a mixed-model accounting for the within-patient correlation.

## Results

### Study population

Baseline characteristics and discontinuations from the ITT population have previously been reported. In brief, most patients were White (93%), female (67%), with HAE type I (87%), and mean (± SD) age of 39.6 (14.9) years. The mean (± SD) time-normalized number of attacks per month during the run-in period was similar in both treatment sequences: 4.6 ± 2.2 for the 40 IU/kg treatment sequences and 3.9 ± 2.0 for the 60 IU/kg treatment sequences.

### Pre-specified subgroup analyses

**Fig. 1:**
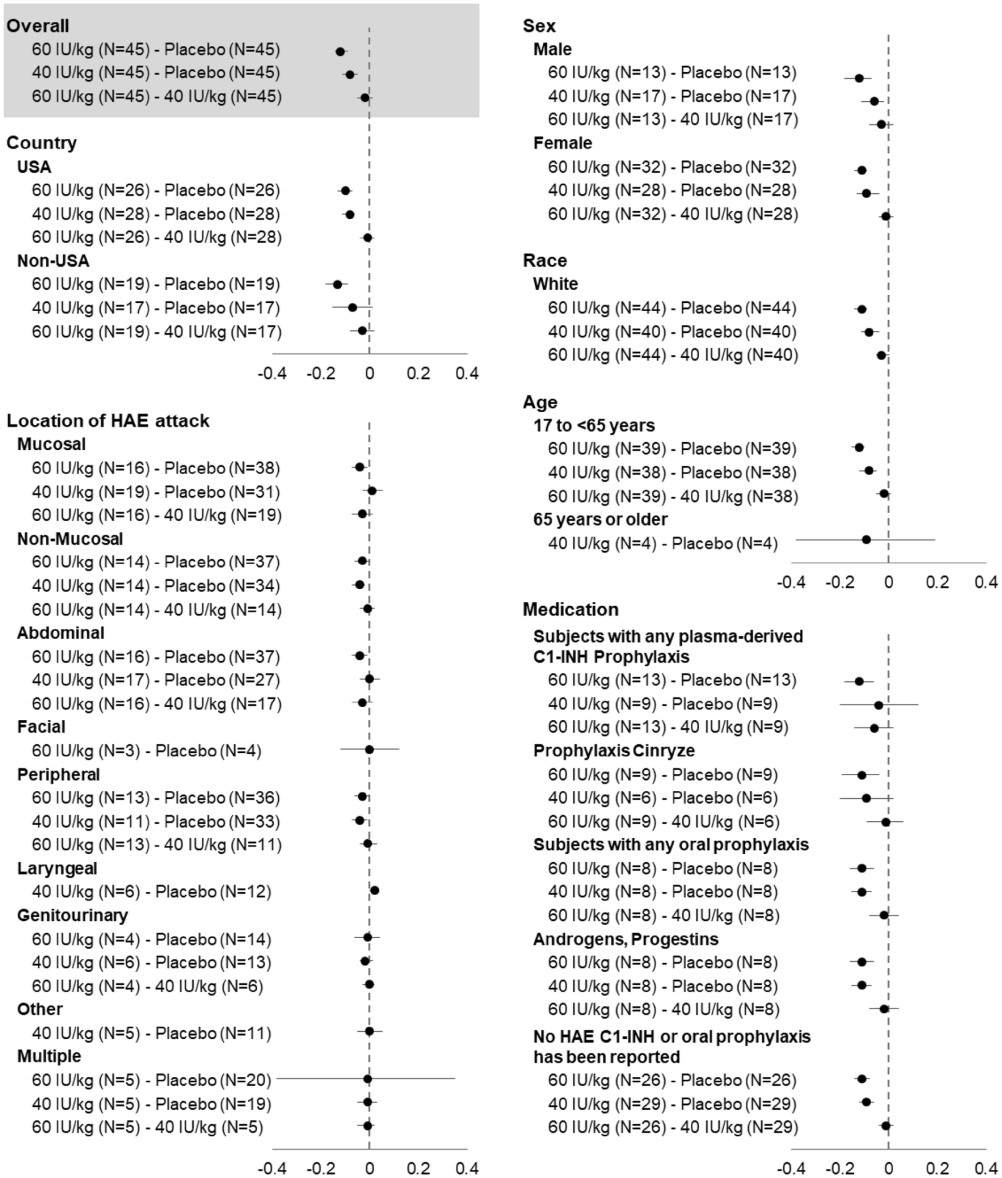
Absolute difference in time-normalized number of HAE attacks (number/day) during treatment with C1-INH (SC) by subgroup. C1-INH: C1-esterase inhibitor; HAE: hereditary angioedema; N: number of patients; SC: subcutaneous; USA: United States of America

Subgroup results for the time-normalized number of HAE attacks are displayed in Fig. 1 and were found similar to the overall analysis results, i.e., the rate of attacks was lower on C1-INH (SC) than on placebo. Similarly, subgroup results for the percentage of responders were also similar to the overall analysis results, i.e., the percentages of responders on 60 IU/kg (36/40 patients, 90%) and on 40 IU/kg C1-INH (SC) (32/42 patients, 76%). The majority of patients were White (84/90 patients, 93.3%), which precluded meaningful assessments by race. C1-INH (SC) was effective in reducing attacks at any body location and in treating patients with any type of prior prophylaxis used before the COMPACT trial.

### Pre-specified exploratory analyses on endpoints including attack severity

Table 2 shows all exploratory endpoints that were not previously reported. The median (range) duration of HAE attacks per patient was 1.57 (1.0, 5.8) days for 40 IU/kg, 1.71 (1.0, 7.0) days for its corresponding placebo, 1.0 (1.0, 5.5) days for 60 IU/kg, and 1.45 (1.0, 4.0) days for its corresponding placebo. Both doses of C1-INH (SC) reduced the time-normalized sum of symptom severity scores relative to placebo.

**Table 2:** Summary of pre-specified exploratory and post hoc endpoints

	40 IU/kg (N = 45)		60 IU/kg (N = 45)	
	C1-INH (SC)	Placebo	C1-INH (SC)	Placebo
Primary efficacy endpoint				
Number of time-normalized HAE attacks/month^a^—mean (95% CI)	1.19 (0.54, 1.85)	3.61 (2.96, 4.26)	0.52 (0.00, 1.04)	4.03 (3.51, 4.55)
Secondary efficacy endpoint				
Uses of rescue medication/month^a^—mean (95% CI)	1.13 (− 1.44, 3.69)	5.55 (3.10, 8.00)	0.32 (− 0.33, 0.97)	3.89 (3.23, 4.55)
Pre-specified exploratory endpoints				
Time-normalized sum of symptom severity scores				
Mean (SD)	0.10 (0.19)	0.44 (0.40)	0.07 (0.15)	0.45 (0.33)
Median (range)	0.02 (0, 0.9)	0.36 (0, 2.0)	0.01 (0, 0.9)	0.38 (0, 1.5)
N	43	44	43	42
Time-normalized sum of symptom severity scores combined with rescue medication use				
Mean (SD)	0.17 (0.35)	0.73 (0.69)	0.09 (0.18)	0.73 (0.51)
Median (range)	0.04 (0, 1.8)	0.59 (0, 3.5)	0.02 (0, 0.9)	0.65 (0.1, 2.4)
N	43	44	43	42
Duration of HAE attacks per patient, days				
Mean (SD)	1.80 (1.08)	2.08 (1.21)	1.58 (0.98)	1.64 (0.66)
Median (range)	1.57 (1.0, 5.8)	1.71 (1.0, 7.0)	1.0 (1.0, 5.5)	1.45 (1.0, 4.0)
Post hoc analysis of treated versus untreated HAE attacks				
Number (%) of patients with HAE attacks	26 (57.8)	40 (88.9)	25 (55.6)	42 (93.3)
Number (%) of patients without HAE attacks	17 (37.8)	4 (8.9)	18 (40.0)	0
Number (%) of patients with missing data about occurrence of HAE attacks	2 (4.4)	1 (2.2)	2 (4.4)	3 (6.7)
Number of all HAE attacks	145	503	71	472
Number (%) of HAE attacks treated with rescue medication	99 (68.3)	421 (83.7)	35 (49.3)	358 (75.8)
With 1 rescue medication	92 (92.9)	375 (89.1)	35 (100)	318 (88.8)
C1-INH (IV) Berinert^®^	83 (90.2)	338 (90.1)	28 (80.0)	271 (85.2)
C1-INH (not Berinert IV)	0	1 (0.3)	0	5 (1.6)
Icatibant (Firazyr^®^)	8 (8.7)	58 (15.5)	7 (20.0)	63 (19.8)
Other	1 (1.1)	11 (2.9)	0	1 (0.3)
With 2 rescue medications	7 (7.1)	33 (7.8)	0	27 (7.5)
C1-INH (IV) Berinert	7 (100)	14 (42.4)	0	6 (22.2)
C1-INH (not Berinert IV)	0	0	0	0
Icatibant (Firazyr)	0	10 (30.3)	0	17 (63.0)
Other	0	1 (3.0)	0	0
With ≥ 3 rescue medications	0	13 (3.1)	0	16 (3.6)
C1-INH (IV) Berinert	0	5 (38.5)	0	2 (15.4)
C1-INH (not Berinert IV)	0	0	0	1 (7.7)
Icatibant (Firazyr)	0	4 (30.8)	0	7 (53.8)
Other	0	0	0	0

C1-INH: C1-esterase inhibitor; CI: confidence interval; HAE: hereditary angioedema; IV: intravenous; N: number of patients in the intention-to-treat population; n: number of patients with data; SC: subcutaneous; SD: standard deviation

^a^Least squares estimate from a mixed-model

### Post hoc analysis of treatment responses in the first 2 weeks of treatment

In the COMPACT study, the first 2 weeks of each treatment period were excluded from the efficacy analysis to account for possible wash-in/wash-out effects, a methodological aspect of a crossover study. In the present post hoc analysis, we evaluated the onset of the prophylactic effect of C1-INH (SC) within the first 2 weeks of treatment initiation. In the first 2 weeks, 12/43 patients (28%) experienced attacks with 40 IU/kg versus 39/44 patients (89%) with corresponding placebo, and 10/43 patients (23%) with 60 IU/kg versus 34/42 patients (81%) with placebo (Table 3). Of the total number of 14 attacks that occurred during the first 2 weeks of C1-INH (SC) 60 IU/kg, 9 (64%) were mild and 5 (36%) were moderate. Of note, no severe attacks occurred during the first 2 weeks of C1-INH (SC) 60 IU/kg prophylaxis (Table 3), whereas of the 70 attacks that occurred under placebo, 22 (31%) were mild, 31 (44%) were moderate, and 17 (24%) were severe. Of the total number of 27 attacks that occurred during the first 2 weeks of C1-INH (SC) 40 IU/kg, 9 (33%) were mild, 10 (37%) were moderate, and 8 (30%) were of severe intensity, whereas of the 78 attacks that occurred under corresponding placebo, 16 (21%) were mild, 39 (50%) were moderate, and 23 (29%) were severe.

**Table 3:** Preventive effect of C1-INH (SC) within 2 weeks of treatment initiation

	40 IU/kg (N = 45)		60 IU/kg (N = 45)	
	C1-INH (SC)	Placebo	C1-INH (SC)	Placebo
Number of patients	43	44	43	42
Number of patients with HAE attacks (%)	12 (28)	39 (89)	10 (23)	34 (81)
Number of HAE attacks	27	78	14	70
Number (%) of HAE attacks by severity				
Mild	9 (33)	16 (21)	9 (64)	22 (31)
Moderate	10 (37)	39 (50)	5 (36)	31 (44)
Severe	8 (30)	23 (29)	0	17 (24)

C1-INH: C1-esterase inhibitor; HAE: hereditary angioedema; N: number of patients in the intention-to-treat population; SC: subcutaneous

### Post hoc analysis of treated versus untreated attacks

Further post hoc analyses of attacks requiring rescue medication are displayed in Table 2. A total of 1191 attacks were recorded across the treatments, mainly with placebo (975 attacks). 913 attacks were treated with rescue medications. The need for rescue medications was approximately tenfold lower with 60 IU/kg (35 treated attacks) versus placebo (358 treated attacks), and fourfold lower with 40 IU/kg dose (99 treated attacks) versus placebo (421 treated attacks). In total, 820 out of 913 attacks (90%) and all treated attacks on 60 IU/kg C1-INH (SC) were treated with only 1 injection of any rescue medication; 5% of the 761 attacks treated with C1-INH concentrate (IV) required ≥ 2 doses, and 22% of the 174 attacks treated with icatibant required ≥ 2 doses. Only attacks in the placebo period required treatment with 3 or more doses of a rescue medication per attack. Higher severity of attacks was associated with a greater percentage of attacks requiring rescue medications (mild 59%, moderate 79%, and severe 92%).

### Responder analysis of patients treated with 60 IU dose

With the approved dosing of 60 IU, 90% of patients were classified as responders, and 4 patients (1 male and 3 female) were classified as non-responders. We did a qualitative post hoc explorative analysis of these cases to assess their treatment outcomes. Clinical findings (number of attacks, HAE symptoms days, and rescue medication use) as well as biochemical findings (C1-INH activity and C4 levels) are presented in Additional file 1: Table S1. All 4 individuals treated with 60 IU/kg had a clinically relevant prophylactic treatment response as it was evident based on a lower observed number of severe attacks, fewer HAE symptom days, and considerably less use of rescue medication with C1-INH (SC) compared to placebo.

## Discussion

The most recent international HAE management guidelines recommend that all patients should be evaluated for consideration of long-term (routine) prophylaxis at each consecutive clinical visit [8]. Additionally, human, plasma-derived C1-INH was recommended as a first-line therapeutic option for prophylactic treatment against HAE attacks. Human C1-INH (IV) (Cinryze), which was Food and Drug Administration (FDA)-approved in 2008, was the first C1-INH product specifically indicated for routine prophylaxis. This treatment demonstrated a 50% reduction of attacks versus placebo and represented a first step in advancement in HAE management [9]. The next major advancement was the first formulation of a human C1-INH for SC administration (C1-INH [SC]; HAEGARDA), which was FDA-approved in June 2017 for routine prevention of HAE attacks. C1-INH(SC) at the currently approved dosing significantly reduced the HAE attack rate by a median of 95% and reduced the need for rescue medication by 100% and also improved patients’ quality of life compared to placebo in the phase 3 COMPACT study [1, 5].

In our analysis we did not see a difference in the prophylactic effect within any patient subgroup investigated. All patients experienced a lower rate of attacks with C1-INH (SC) compared to placebo and C1-INH (SC) was effective in preventing attacks at any body location and in patients with any type of prior prophylaxis used.

Our exploratory and post hoc analyses demonstrate that C1-INH (SC) reduces the attack number and attack severity compared to placebo as well the need for any use of HAE rescue medication. C1-INH (SC) prophylaxis effectively reduced the need for rescue medications in patients with type I or II HAE in a dose-dependent manner (tenfold with a 60 IU/kg dose and fourfold with a 40 IU/kg dose) compared to placebo.

A practical question concerns how soon after initiation of C1-INH (SC) prophylaxis the preventive effect first becomes evident. When designing the pivotal study, it was estimated that it would take about 4 half-lives to achieve steady-state serum levels of functional C1-INH which would be achieved after 2 weeks of treatment initiation. We explored the onset of the preventive effect of C1-INH (SC) after treatment initiation, and found that it is evident already within the first 2 weeks after treatment, as evidenced by fewer HAE attacks and fewer attacks of severe intensity.

With the approved dosing regimen of 60 IU/kg, we noted that 4 out of 45 patients had a reduction of less than 50% in attack frequency, a criterion that has been used to define a responder to HAE prophylactic treatment. When we explored broader patient relevant outcomes such as symptom days, severity of attacks, and use of rescue medication, we noticed a clinically meaningful treatment benefit in all 4 patients. These findings suggest that an outcome assessment based on a single attack rate value alone may not be adequate and sensitive enough. We suggest more research is needed in refining definitions of most appropriate HAE outcomes.

Several limitations must be considered when interpreting the results of this study. The subgroups in our pre-specified analyses were small due to the fact that HAE is a rare disease. Also, the post hoc analyses presented here are not considered confirmative. In addition, since the data were from a clinical study with an efficacy observation period of 14 weeks, effects observed may not necessarily apply to a patient being treated over a longer period of time.

## Conclusions

Our analyses provide additional evidence that C1-INH (SC) prophylaxis is effective in reducing the number of angioedema attacks independent of any baseline patient characteristics. Current data suggest that C1-INH (SC) can provide substantial and clinically meaningful relief from disease and treatment burden for all patients with type I or II HAE. The preventive effect of C1-INH (SC) is already evident within 2 weeks of treatment initiation.

## Supplementary information

**Additional file 1: Table S1.** Patients A–D with <50% reduction in HAE attack rate treated with 60 IU/kg C1-INH (SC).

**Additional file 2.** COMPACT Study Committees, Investigators, and other Collaborators.

## Data Availability

All data are contained in the paper.

## Abbreviations

C1-INH: C1-esterase inhibitor
C1-INH (SC): subcutaneous C1 inhibitor
FDA: Food and Drug Administration
HAE: hereditary angioedema
ITT: intention-to-treat
IV: intravenous
SD: standard deviation
TP1: treatment period 1
TP2: treatment period 2
US: United States
