# Supplementary material for: Subcutaneous C1 inhibitor for prevention of attacks of hereditary angioedema: additional outcomes and subgroup analysis of a placebo-controlled randomized study
Source: Allergy Asthma Clin Immunol. 2019 Aug 28;15:49. doi: 10.1186/s13223-019-0362-1 (PMC6714075; doi:10.1186/s13223-019-0362-1)
Supplement: Supplementary file 1 — Additional file 1: Table S1. Patients A–D with <50% reduction in HAE attack rate treated with 60 IU/kg C1-INH (SC). [file 13223_2019_362_MOESM1_ESM.docx]

Table S1 Patients A – D with <50% reduction in HAE attack rate treated with 60 IU/kg C1-INH (SC)

| **Study period** | **Prior to study  (3 months)** | **C1-INH (SC) (16 weeks)** | **Placebo (16 weeks)** |
| --- | --- | --- | --- |
| **Patient A** |  |  |  |
| Number of attacks  (mild/moderate/severe) | 4 | 9 (6/2/1) | 12 (1/2/9) |
| HAE rate attacks/month | 1.3 | 2.2 | 3.4 |
| HAE symptoms days/month | - | 6.6 | 10.1 |
| Number of rescue medications (medication used) | - | 1 (Icatibant) | 17  (Icatibant, Berinert, other C1-INH) |
| C1-INH activity level (%) | 8 | 76 | 22 |
| C4 level (mg/mL) | 6 | 21 | 6 |
| **Patient B** |  |  |  |
| Number of attacks  (mild/moderate/severe) | 14 | 2 (2/0/0) | 3 (2/1/0) |
| HAE rate attacks/month | 4.7 | 0.6 | 0.9 |
| HAE symptoms days/month | - | 0.3 | 0.9 |
| Number of rescue medications (medication used) | - | 1 (Berinert) | 3 (Berinert) |
| C1-INH activity level (%) | 2 | 81 | 26 |
| C4 level (mg/mL) | 9 | 18 | 5 |
| **Patient C** |  |  |  |
| Number of attacks  (mild/moderate/severe) | 5 | 5 (0/5/0) | 7 (4/3/0) |
| HAE rate attacks/month | 1.7 | 1.1 | 1.9 |
| HAE symptoms days/month | - | 1.5 | 4.1 |
| Number of rescue medications (medication used) | - | 4 (Berinert) | 5 (Berinert) |
| C1-INH activity level (%) | 31 | 108 | 23 |
| C4 level (mg/mL) | 18 | 19 | 9 |
| **Patient D** |  |  |  |
| Number of attacks  (mild/moderate/severe) | 17 | 7 (1/6/0) | 8 (2/3/3) |
| HAE rate attacks/month | 5.7 | 2.2 | 1.7 |
| HAE symptoms days/month | - | 1.6 | 13.7 |
| Number of rescue medications (medication used) | - | 1 (Icatibant) | 6 (Berinert, Icatibant) |
| C1-INH activity level (%) | 13 | 24 | 13 |
| C4 level (mg/mL) | 7 | 11 | 3 |

C1-INH = C1-esterase inhibitor, HAE = hereditary angioedema, SC = subcutaneous.

C1-INH functional activity and C4 protein level values displayed are at screening prior the study, and last available measurement time point during placebo and active treatment period. C1-INH activity was assessed by a validated chromogenic assay (Berichrom C1-INH, Siemens; reference range: 70–130% of norm) and C4 protein by nephelometry (C4 reagent, Beckman Coulter; reference range: 10–40 mg/dL).
