# Supplementary material for: Subcutaneous C1 inhibitor for prevention of attacks of hereditary angioedema: additional outcomes and subgroup analysis of a placebo-controlled randomized study
Source: Allergy Asthma Clin Immunol. 2019 Aug 28;15:49. doi: 10.1186/s13223-019-0362-1 (PMC6714075; doi:10.1186/s13223-019-0362-1)
Supplement: Supplementary file 2 — Additional file 2. COMPACT Study Committees, Investigators, and other Collaborators. [file 13223_2019_362_MOESM2_ESM.docx]

Additional file

**COMPACT Study Committees, Investigators, and other Collaborators**.

Steering Committee: Bruce L. Zuraw (Chair), Marco Cicardi, Timothy Craig, Hilary Longhurst

Data and Safety Monitoring Board: Clive Grattan (Chair), Konrad Bork, Wolfhart Kreuz

CSL Behring, Marburg, Germany and King of Prussia, PA, USA (Sponsor): Jonathan Edelman, Debra Bensen-Kennedy, Ingo Pragst, Michael Lai, Peter Polos, Debora Williams-Herman, Sylvia Herget, Patel Gaurang, Tanja Rosenberg, Corinne Clement, Dipti Pawaskar, Ridhi Parasrampuria, Ying Zhang, Thomas Machnig, Hanno Waldhauser, Henrike Feuersenger, John-Philip Lawo, Doris Lang, Sylvia Hoernlein, Jutta Miller, Martin Millberger, Kirstin Foeller, Ferdinande Ellis, Daniel Wood, Andrew Meads, Michele Walsh, Zak Huang, Karin Mueller-Stark, Annette Feussner, Uwe Kalina, Iris Jacobs, Sylvia Hoernlein, Xiang Ma, Shanta Rigsby, Elke Scheffler, Frank Schaller, Jan-Friedrich Fischer, Christiane Kolb

Medical Monitoring: INC Research, LLC Raleigh, US

Biostatistics Analysis: PAREXEL International, Berlin, Germany

Central Clinical Laboratory: ACM Medical Laboratory, Rochester, US

Bioanalytical / Pharmacokinetic Analysis: CSL Behring GmbH, Marburg, Germany

Clinical Supply Service: Catalent Pharma Solutions, Schorndorf, Germany

Interactive Response Technology System: Perceptive eClinical Limited, Nottingham, UK

Data Management: Chiltern International GmbH, Bad Homburg, Germany

Electronic Diary: CRF Health, PA, US

Safety Monitoring & Pharmacovigilance: CSL Behring GmbH, Marburg, Germany

**Principal Investigators (in bold)** and supporting personnel:

*Australia*: Campbelltown: **C.H. Katelaris**, B. Frankum, P. Burton, F. Perram, K. Bennett, K. Keat; *Canada*: Toronto: **G.L. Sussman**, G. Levi, W. Gould, Hamilton: **P.K. Keith**, P. Ferrie, S. Waserman, Ottawa: **W. Yang**, A. Crawley, S. Santucci, J. O’Quinn, Quebec: **J. Hebert**, S. Claude, L. Potvin, A. Perron, M. Ouellet, R. Gagnon, Alberta: **B. Ritchie**, J. Skripitsky, R. Doepker, J. Jabs; *Czech Republic*: Hradec Kralove: **P. Kralickova**, I. Krcmova, I. Tepla, R. Martincova, M. Novosadova, R. Vojtechova, Pilsen: **J. Hanzlikova**, R. Matouskova, M. Vachova, M. Dockal; *Hungary:* Budapest: **H. Farkas**, L. Varga, N. Veszeli, G. Temesszentandrási, K.V.Kőhalmi, L. Egri, B. Csonka, J. Bali; *Israel:* Tel Hashomer: **A. Reshef**, I. Leibovich, R. Ofir, M. Kidon-Yankovich, S. Fridland, A. Merlinsky, V. Leibo, Tel Aviv: **S. Kivity**, D. Dror, L. Kadar, S. Benor, S. Beer, R. Labrisch, M. Yehuda; *Italy*: Milano: **M. Cicardi**, E. Bonanni, M. Wu, A. Zanichelli, M. Mansi, F. Sechi, Catania: **S. Neri**, A. Rizzotto, F. Giardino, D. Maiorca, F. Fidone, M. Amico-Roxas; *Romania*: Cluj: **I. Crisan**, M. Varga, M. Iftene, E. Buzdugan, I.D. Badiu-Tisa, A. Pop; *Spain*: Barcelona: **M. Guilarte**, A Sala, P. Suné, V. Cardona, N. Moreno, M. Labrador, Madrid: **T. Caballero**, P. Gómez Salcedo, R. Cabañas, M. Pedrosa, D. Rivero-Paparoni, P. Palao-Ocharán, C. Gómez-Traseira, A. Alvez, E. Phillips, A. Borobia, **M.L. Baeza**, A. Prieto, M. Angel Conejo, A. Mur, J.M. Zubeldia, Valencia: **M.D. Hernandez**, M. Tordera, E. Ibanez, E. Gimeno; *United Kingdom*: Brighton: **M. Tarzi**, M. Flowerdew, A. Frew, K. Davies, K. Nambiar, R. Rye, D. Lambert, R. Chowdhry, R. Varghese, A. Murray, L.S. Robinson, C. McPherson, A. Beddoe, London: **H. Longhurst**, R. Sarpong, A. Kieliszkowska, S. Grigoriadou, M-J. Valda, G. Boyapati, A. Zdanaviciene, D. Andrews, J. Laffan, I. Nasr, A. Bellin, B. Crone, C. Evans, A. Twumasi; *United States*: Hershey: **T. Craig**, C. Mende, S. Cleary, M. Stanton, C. Schaeffer, G. Ghaffari, F. Ishmael, E. Rael, N. Vernon, N. Kalra, J. Zaragoza-Buxo, V. Reddy, L. Buyantseva, A. Fazzi, H. Heisey, J.A. Brown, A. Kane, M. Sweigart, Chevy Chase: **H.H. Li**, T. Johnson, L. Kosh, S. Shaikh, M. Scarupa, A. Economides, M. White, M. Kaliner, Lake Oswego: **J. Baker**, S. Persons, A. Newman, M.J. Noonan, A. Johnson, Dallas: **W.R. Lumry**, J. Tucker, D. Aguilar, Cincinnati: **J. Bernstein**, K. Murphy, B. Berendts, K. Klefas, S. Merritt, Toledo: **S.M. Rehman**, P Bak, R Chambers, Boston: **A. Banerji**, M Iandoli, M Canova, S Bednar, J Vetrano,Tulsa: **I. Hussain**, M. Crawford, S. Poston, Birmingham: **J. Bonner**, D. Paige, K. Fuqua, K. Hale, H. Combs, H. Haggard, K. Phillipson, **J. Anderson**, W. Soong, M. Sikora, M. Lemke, P. Luthin, C. Neal, Richmond: **L.B. Schwartz**, E. Gilbert, K. Bradley, W. Zhao, M. Abdalgani, A.M. Irani, B. Ward, A. Perry, A. Hill, S. Twombly, Spokane: **R. Gower**, C Witte, J Rud,S Levitch, T Vonasek, Walnut Creek: **J. Jacobs**, J. Curl, K. Silva, T. Mostofi, C. Doss, N. Schultz, Columbus: **D. McNeil**, A Thornton, A Urbank, P Rancitelli, N Palecek, T Singletary, Colorado Springs: **R. Nathan**, J. Altman, E. Soltero, K. Soltero, D.F. Soteres, L.M. Webb, D.K. Walters, J.A. Allen, Bell Gardens: **G.N.Salem**, E. De La Cruz, A. Hafez, Scottsdale: **M.E. Manning**, T.J. Tyma, L. Endean, A. Tolman, M. Naggs, A. Davis, J. Nelson, M. Parks, Orange: **D.S. Levy**, B. Borts, La Jolla: **M. Riedl**, J. Blair, B. Zuraw, J. Sun, Y. Brown, B. Daly, C. Tanguilig, T. Le, M. Tutto, M. Berg, M. LeFebvre, M. Mallari
